# Health professionals in Flanders perceive the potential health risks of vaping as lower than those of smoking but do not recommend using e-cigarettes to their smoking patients

**DOI:** 10.1186/s12954-016-0111-4

**Published:** 2016-06-24

**Authors:** Dinska Van Gucht, Frank Baeyens

**Affiliations:** Thomas More University College Antwerp and KU Leuven, Antwerp, Belgium; KU Leuven, Leuven, Belgium

**Keywords:** Electronic cigarette, Risk perception, Attitudes

## Abstract

**Background:**

Many misperceptions of both risks and opportunities of e-cigarettes (e-cigs) exist among the general population and among physicians, although e-cigs could be a valuable harm reduction tool for current smokers.

**Methods:**

Two groups in Flanders, namely general practitioners (GPs; family doctors) and tobacco counselors filled out an online questionnaire with regard to their attitudes and risk perceptions concerning e-cigs. Statements included were on the safety and the addictive properties of e-cigs in absolute terms, whereas other items compared e-cigs with regular tobacco cigarettes. Statements about possible “gateway” and “renormalization” effects, selling to minors, and use in public places and on the potential of e-cigs as a smoking cessation aid were also included. Respondents were also asked for the rate at which their patients asked information about e-cigs, if they would recommend e-cigs to their smoking patients, and whether they had information brochures on e-cigs.

**Results:**

About 70 % believed that e-cigs are harmful to vapers, and about half to two thirds believed that e-cigs are carcinogenic, increase cardiovascular risk, and increase the risk of chronic lung disease. Also, a substantial minority incorrectly believed these risks to be no less than those resulting from regular smoking. Ten to almost 20 % disagreed that e-cigs are healthier and represent less risk for the main serious smoking-related diseases than conventional cigarettes. More than half of the respondents disagreed that e-cigs are an effective smoking cessation aid. None (0 %) offered the strongest level of agreement for recommending e-cigs to their clients/patients, but GPs agreed to a lesser degree a bit more often than tobacco counselors. Almost none had information leaflets for potentially interested patients. Finally, the majority of our sample also believed that e-cigs will cause renormalization of smoking and that e-cigs will lead to an uptake of conventional smoking and disagreed with allowing vaping in enclosed public places.

**Conclusions:**

Health professionals in Flanders perceive the potential health risks of vaping as lower than those of smoking but do not recommend using e-cigs to their smoking patients.

## Background

Despite all tobacco control efforts, reductions in smoking prevalence in Belgium—much like in most other countries in Western Europe—appear to have stalled over the last decade [1]. Yearly surveillance of smoking among samples that are representative of the Belgian population (aged 15–75) shows that in the most recent period 2011–2015, smoking prevalence kept hovering around 25 %. Many smokers claim to have the intention to quit and many make actual quit attempts, but the vast majority are unsuccessful. Of those who choose to try quitting without any assistance (by “willpower” alone), no more than 3–5 % are typically found to be abstinent 6–12 months later [2]. Those smokers who choose medically approved smoking cessation aids, including quit-smoking medication (such as Varenicline), nicotine-replacement therapy (NRT), and/or behavioral counseling, at best double or triple their chances of long-term success [3]. For example, in the most recent analysis of the UK National Health Stop Smoking Services’ long-term outcomes, no more than 8 % of the clients (most of them having received a combination of behavioral counseling plus NRT or quit-smoking medication) showed carbon monoxide (CO)-validated cessation at 1 year [4].

This failure of traditional tobacco control may in part be related to the fact that its ultimate objective is the eradication of any form of tobacco and nicotine use. This goal may not be attainable, nor even be desirable for many smokers. Tobacco harm reduction (THR)—encouraging the substitution of low-risk alternatives—may provide a viable alternative for those smokers who cannot or do not want to cease all tobacco and/or nicotine consumption [5–7]. Like other low-risk nicotine products such as smokeless tobacco (e.g., Swedish suns), electronic cigarettes (e-cigs) may represent a useful tool for THR.

E-cigs completely avoid the combustion of organic material (c.q., tobacco), and hence most of the toxic and carcinogenic chemicals that are present in cigarette smoke. A systematic review by Burstyn of current knowledge of the chemical composition and the toxicological profile of e-cig aerosols [8] indicates that the deleterious constituents of tobacco smoke, including carcinogens, are either absent or, if present, at levels mostly below 1 % of the levels typically found in cigarette smoke, whereas the main chemicals predominant in, or unique to, e-cig vapor have not been associated with any serious risk [9]. Burstyn [8] (p. 12) thus concluded that “the current state of knowledge about the chemistry of contaminants in liquids and aerosols associated with electronic cigarettes indicates that there is no evidence that vaping produces inhalable exposures to these contaminants at a level that would prompt measures to reduce exposure by the standards that are used to ensure safety of workplaces.” In the same vein, reviews of the studies on the clinical safety of (short-term) exposure to e-cig aerosols come to the conclusion that there is currently no evidence for (irreversible) harmful effects of using e-cigs on the respiratory or cardiovascular system; that the effects of vaping are beyond any reasonable doubt significantly less harmful than the effects of smoking; and that e-cigs probably also pose no more than minor health risks in an absolute sense. However, given the fact that e-cigs have been widely consumed for less than a decade, there inevitably remains a degree of uncertainty about the health effects of long-term e-cig use. [10, 11], see also [9].

For the e-cig to be useful as a THR tool, it is important to demonstrate not only that it is indeed a low-risk nicotine delivery product but also that it is accepted by (current) smokers and that it is effective with respect to smoking reduction or cessation. Initial evidence bearing on these questions is promising. E-cigs seem to gain wide acceptance and substantial market penetration among smokers, at least in those countries where e-cigs with nicotine are easily available. According to the latest Eurobarometer [12], about 10 % of the total EU population aged 15+ (about 43 million people) are smokers or ex-smokers that use or have ever tried e-cigs. Of those, 14 % report complete smoking cessation and 21 % report smoking reduction, whereas 13 % report having quit smoking but started again. In absolute numbers, these Eurobarometer figures translate into roughly 6 million quitters, 9 million reducers, and 5.5 million “temporary quitters” among smokers that use or ever tried e-cigs. In the most recent ASH data [13], about 59 % of all smokers in Great Britain ever tried an e-cig, and 18 % (2.6 million adults) currently use e-cigs (about 2 out of 5 users are now ex-smokers and 3 out of 5 are current smokers or “dual users”), mainly to reduce the amount they smoke (dual users) or to remain abstinent from tobacco smoking (quitters).

There is also evidence that medical professionals and tobacco counselors who choose to intervene to aid smoking cessation can promote e-cigs. This is certainly not trivial: for example, in a recent survey by Phillips (*n* = about 20 000) [14] among American vaping enthusiasts (members of the Consumer Advocates for Smoke-free Alternatives Association (CASAA)), about 1 out of 5 reported either “to have become interested in e-cigs in the first place due to advice of a healthcare provider,” or “that a provider volunteered a recommendation to try e-cigs, though the subject was already using them or considering it.” In a randomized controlled trial (RCT) in New Zealand, by Bullen and colleagues, a comparison was made between the efficacy of e-cigs and nicotine patches for smoking cessation in smokers wanting to quit [15]. After 6 months, 7 % of the participants were completely abstinent from tobacco cigarettes with nicotine e-cigs, 6 % with nicotine patches, and 4 % with “placebo” (no-nicotine) e-cigs. In a second RCT in Italy, Caponnetto and colleagues offered smokers not intending to quit either nicotine-containing e-cigs or no-nicotine e-cigs [16]. After 12 months, quit rates were 11 versus 4 %, respectively. It is important to note that these earlier prospective trials and RCTs used closed system e-cigs that are by now obsolete and underperforming compared with current models. A more recent RCT by Adriaens, Van Gucht, Declerck, and Baeyens [17] and a prospective cohort study by Polosa et al. [18] assessed the efficacy of better performing open system e-cigs in Flemish and Italian (resp.) smokers without the intention to quit and observed biologically verified quit rates in 21–36 % of all participants at 6–8 months after the start of the intervention and a smoking reduction of at least 50 % in an additional 23–30 % of participants. Finally, in another study by Polosa and colleagues targeting a somewhat different population of smokers who were naïve about e-cigs but clearly interested in them, a prospective real-world study of first-time vape shop visitors in Italy, the 12-month quit rate was as high as 41 %, whereas an additional 25 % of customers reduced smoking by at least 50 % [19]. In sum, in several clinical trials and a real-world study, the use of e-cigs has been demonstrated to be associated with smoking cessation and reduction.

However, many misperceptions of both risks and opportunities of e-cigs exist both in the general population and among physicians, and the latter appears to be somewhat reluctant to recommend using them to their patients. So far, one study by Kandra, Ranney, Lee, and Goldstein [20] measured attitudes toward e-cigs among North Carolina physicians treating adult smokers. About two-thirds of the physicians who participated correctly believed that e-cigs lower the risk of cancer when they are used instead of smoking cigarettes, and a similar number agreed that e-cigs are a helpful aid for smoking cessation. Nevertheless, only 35 % actually recommended an e-cig to their smoking patients. In a similar vein, Steinberg, Giovenco, and Delnevo [21] found that two-thirds of a convenience sample of US physicians who participated in a web-based survey on patient-physician communication regarding e-cigs reported that patients inquire about e-cigs, whereas no more than 30 % of the physicians reported that they had recommended e-cigs as a smoking cessation tool. In another study by Pepper, Gilkey, and Brewer, among US pediatricians and family medicine physicians who provide primary care to adolescents [22], only 1 out of 4 would recommend e-cigs to their adolescent patients as a smoking cessation tool. If asked, less than half of these physicians would tell their smoking patients that they believe that e-cigs are less harmful than cigarettes.

Results from a US National Survey 2012–2013 are in line with these observations in physicians: Only 51 % of the general population believed that e-cigs are less harmful than cigarettes [23]; the authors concluded that there is a strong discrepancy between the scientific evidence and the perceptions of the general population when it comes to tobacco harm reduction. The same pattern has been confirmed in Great Britain, the results of the latest Action on Smoking and Health survey [24] showing that only 45 % of the general public believe e-cigs to be less (30 %) or a lot less (15 %) harmful than cigarettes, whereas *among current smokers*, only 12 % believe e-cigs to be a lot less harmful and 25 % believe e-cigs to be more or equally harmful than smoking.

In the current study, we compared two groups of healthcare providers in Flanders with regard to their attitudes and risk perceptions concerning e-cigs, registered tobacco counselors having obtained a training and being certified as “tabacologists” and general practitioners (GPs). As a point of reference, we also report the data obtained in a convenience sample of members from the general public.^1^ Apart from measuring general perception of harm from using e-cigs relative to smoking tobacco cigarettes, we also gained information on their beliefs concerning cancer, cardiovascular, and respiratory risks, and concerning the addictive properties of vaping. We also assessed attitudes about possible “gateway effects” and “renormalization” of smoking, their opinion about selling e-cigs to minors, the use of e-cigs in enclosed public places, and the potential of e-cigs as a smoking cessation tool.

## Methods

### Participants

Tobacco counselors and GPs were contacted through different channels. In 2014, in Flanders, there were 205 registered tobacco counselors who were all invited via e-mail to participate in our study: more than 25 % responded (*n* = 54) to our request. We e-mailed different associations of GPs to distribute our request (via e-mail) to their members, because individual e-mail addresses were not available. We do not know how many of these associations actually forwarded the invitation to participate and consequently, how many GPs received our invitation. The response rate in this population was low (*n* = 22), probably also because of the fact that GPs are overburdened by requests to participate in research. In total, the sample consisted of 76 respondents who completed the questionnaire: 29 % were GPs and 71 % were people trained as tobacco counselors (with another health care profession such as for example psychologists).

### Measures

The online questionnaire, made in Qualtrics, started with some questions assessing background information: age, gender, smoking status (smoker, ex-smoker, non-smoker), vaping status (ever tried an e-cig, regular user of e-cig), profession, number of years in the field, number of patients (in total and per workday), and estimation of the prevalence of smokers among their patients. Respondents were also asked for the rate at which they informed their patients about e-cigs (never, almost never, sometimes, often) and whether they had information brochures on e-cigs (yes-no). The questions on the attitudes and perceptions about e-cigs were based on those used by Kandra et al. [20] but extended with others constructed by the authors (for the literal English translation of all statements that appeared in the questionnaire, see Table 3). The statements could be rated from 1 (totally disagree), over 2 (rather disagree) and 3 (rather agree), to 4 (totally agree). Some were on the safety and the addictive properties of e-cigs in absolute terms, for example “The electronic cigarette is harmful to the vaper” and “The electronic cigarette with nicotine has an addictive effect,” others compared e-cigs with regular tobacco cigarettes, for example “The risk of cardiovascular disease is lower for the electronic cigarette than for the conventional cigarette.” Statements about possible “gateway” and “renormalization” effects, e.g., “I think that the electronic cigarette will lead to uptake of conventional smoking,” selling to minors “I think the electronic cigarette should be prohibited to minors (18 years),” and use in public places “I think the electronic cigarette can be used in public places where smoking is prohibited” were also included. Finally, respondents were also asked about the potential of e-cigs as a smoking cessation aid, if they would support patients who spontaneously tell them they want to start using the e-cig in this decision and if they would recommend e-cigs to their smoking patients (see also Table 3).

### Procedure

In the invitation e-mail, the goal of the study was stated, namely to gain more insight in the knowledge and attitudes regarding e-cigs in different populations. Participants were then referred to the online survey via a Qualtrics link. The procedure was approved by the ethical committee of Thomas More University College.

## Results

### Participants’ characteristics

Respondents averaged 45 years old (range 24–65). Forty percent of the tobacco counselors and 33 % of the GPs were male. Their smoking status appears in Table 1. Three respondents had tried an e-cig, but none used them regularly.

**Table 1:** Participants’ smoking status

	Smoker (%)	Ex-smoker (%)	Non-smoker (%)
GPs	5	18	77
Tobacco counselors	0	43	57
Total	1	36	63

The respondents had on average more than 17 years experience in the field, more than 800 patients in total, and saw about 12 patients on a regular workday (Table 2). They estimated that 42 % of their patients/clients were current smokers. Note that tobacco counselors estimated that among their patients, only half smoke this is because most tobacco counselors also have another profession (nurse, psychologist, etc.).

**Table 2:** Participants’ characteristics

	GPs	Tobacco	Total
	M	M	M
Number of years in field	21	16	18
Number of patients in total	1693	263	808
Number of patients/workday	19	9	12
Estimated % of smoker among patients	25	51	42

Only two professionals *never* or *seldom* asked their patients/clients about their current smoking status. Almost one third asked it *sometimes*, and two thirds asked it *always*. For obvious reasons, tobacco counselors asked this significantly more frequently than GPs, Ws = 1884, *z* = −2.701, *p* < .01. The majority reported that their patients inform about e-cigs, significantly more so for tobacco counselors than for GPs, Ws = 551, z = −3.684, *p* < .001; a quarter of professionals reported that their patients do it *often*, about half *sometimes*, and the other quarter *almost never* to *never*. Finally, only five professionals answered affirmatively that they had any information brochures on vaping to hand out to their patients.

### Perceptions and attitudes on e-cigs

In this section, we take a closer look at the GPs’ and tobacco counselors’ perceptions and attitudes on e-cigs. We refer to Table 3 for the distributions of responses for each statement.

**Table 3:** Tobacco counselors and general practitioners’ distributions of responses on the different statements

	Totally disagree (%)		Rather disagree (%)	Rather agree (%)	Totally agree (%)
The e-cig is harmful to the vaper	TCs	4	26	63	7
	GPs	0	32	55	14
	Total	3	28	61	9
The e-cig is harmful to people in the vicinity of the vaper	TCs	22	48	24	6
	GPs	9	55	27	9
	Total	18	50	25	7
The e-cig is healthier than the conventional cigarette	TCs	7	11	39	43
	GPs	5	9	55	32
	Total	7	11	43	40
The e-cig is carcinogenic	TCs	11	41	35	13
	GPs	18	41	27	14
	Total	13	41	33	13
The risk of cancer is lower for the e-cig than for the conventional cigarette	TCs	2	13	43	43
	GPs	5	5	59	32
	Total	3	11	47	40
The e-cig increases the cardiovascular risk	TCs	6	30	44	20
	GPs	9	23	55	14
	Total	7	28	47	18
The risk of cardiovascular disease is lower for the e-cig than for the conventional cigarette	TCs	7	7	52	33
	GPs	5	5	73	18
	Total	7	7	58	29
The e-cig increases the risk of chronic lung disease	TCs	11	41	41	7
	GPs	9	41	36	14
	Total	11	41	40	9
The risk of chronic lung disease is lower for the e-cig than for the conventional cigarette	TCs	4	17	44	35
	GPs	5	9	73	14
	Total	4	15	53	29
Nicotine is a very harmful component of the e-cig	TCs	24	30	28	19
	GPs	4	50	27	18
	Total	18	36	28	18
The e-cig with nicotine has an addictive effect	TCs	2	7	35	56
	GPs	0	14	41	46
	Total	1	9	37	53
The e-cig without nicotine has an addictive effect	TCs	20	43	32	6
	GPs	18	46	32	5
	Total	20	43	32	5
I think the e-cig can be used in public places where smoking is prohibited	TCs	70	19	9	2
	GPs	36	36	18	9
	Total	61	24	12	4
I think the e-cig should be prohibited to minors (18 years)	TCs	2	11	20	67
	GPs	5	5	27	64
	Total	3	9	22	66
I think the e-cig will cause renormalisation of smoking	TCs	2	15	50	33
	GPs	5	23	46	27
	Total	3	17	49	32
I think that the e-cig will lead to uptake of conventional smoking	TCs	4	22	52	22
	GPs	9	27	46	18
	Total	5	24	50	21
The e-cig is an effective smoking cessation aid	TCs	24	43	32	2
	GPs	5	46	50	0
	Total	18	43	37	1
I support patients, who spontaneously tell me they want to start using the e-cig, in this decision	TCs	20	39	33	7
	GPs	5	27	59	9
	Total	16	36	41	8
I recommend the e-cig to my patients	TCs	57	37	6	0
	GPs	32	59	9	0
	Total	50	43	7	0

*TCs* tobacco counselors, *GPs* general practitioners

More than two thirds of the sample studied agreed (“rather agree” and “totally agree combined) with the statement that e-cigs are harmful to the vaper, and about one third agreed that e-cigs are harmful to people in the vicinity of the vaper. Almost 1 out of 5 of the professionals disagreed (“rather disagree” and “totally disagree” combined) that e-cigs are healthier than conventional cigarettes. About half of the professionals agreed with the statement that e-cigs are carcinogenic, while more than 8 out of 10 agreed, however, that the risk of cancer is lower for e-cigs than for the conventional cigarette. According to two thirds of our sample, e-cigs increase cardiovascular risk, but more than 8 out of 10 agreed with the statement that the risk of cardiovascular disease is lower for e-cigs than for conventional ones.

With regard to chronic lung disease, almost half of the professionals agreed that e-cigs increase that risk, but again, about 8 out of 10 agreed with the statement that the risk of chronic lung disease is lower for e-cigs than for conventional cigarettes. About half of the sample agreed with the statement that nicotine is a very harmful component of e-cigs. With regard to the addictive properties of e-cigs, 9 out of 10 agreed that e-cigs with nicotine have an addictive effect, and more than one third agreed that e-cigs without nicotine have an addictive effect.

More than 8 out of 10 of the respondents disagreed with allowing the use of e-cigs in enclosed public places where smoking is prohibited. Almost 9 out of 10 agreed that e-cigs should be prohibited to minors and 8 out of 10 agreed that e-cigs will cause renormalization of smoking; moreover, 7 out of 10 believed that e-cigs will lead to uptake of conventional smoking.

About two thirds of the respondents disagreed with the statement that e-cigs are an effective smoking cessation aid, with a trend for tobacco counselors to answer more negatively than GPs (24 vs. 5 % *totally disagree*, see Table 3). In order to test the reliability of this interesting difference between the two populations in the endorsement of the different response categories for this item, we used the Wilcoxon rank-sum test, Ws = 1932, *z* = −1.813, *p* = .07.

About half of the respondents support patients who spontaneously tell them that they want to start using e-cigs; noteworthy, GPs were significantly more supportive than tobacco counselors (68 vs. 41 %), Ws = 1901.5, *z* = −2.163, *p* < .05. When professionals were asked if they would recommend e-cigs to their smoking patients, none *totally agreed* and less than 1 out of 10 *rather* agreed, while 4 out of 10 *rather disagreed* and 5 out of 10 *totally disagreed*. In line with the above, tobacco counselors answered significantly more negatively than GPs (57 vs. 32 % *totally disagree* with recommendation to the patients, see Table 3), Ws = 1926, *z* = −1.916, *p* = .05.^1^

## Discussion

In this survey of health professionals’ beliefs about e-cigs, striking similarities with earlier results obtained in the UK and the USA (also see [25]) were observed. There is currently no identified serious health risk associated with vaping, and the likelihood of long-term e-cig use being implicated in the development of chronic or fatal disease is estimated to be low and in the range of 1/20th to 1/100th of that of smoking [8–11]. About 3 in 4 of our studied sample believed that e-cigs are harmful to vapers, whereas around half to two thirds of the sample believed that e-cigs are carcinogenic and increase the cardiovascular risk and the risk of chronic lung disease. Also, a substantial minority incorrectly believed these risks to be no less than those resulting from regular smoking: a majority of the professionals rightfully believed that vaping involves smaller health risks than smoking, but 10–20 % of them disagreed that e-cigs are healthier and represent less risk for the main serious smoking-related diseases than conventional cigarettes.

Whereas the interpretation of participants’ answers on these items asking about the perceived *relative risks* of e-cigs (compared to smoking) is rather straightforward, the meaning of the answers on the items asking for the perceived harmful effects of e-cigs *in absolute terms* may be more problematic, especially considering the nature of the response alternatives provided (from *totally disagree* to *totally agree*). Namely, several different interpretations of the latter questions and/or responses alternatives may have been possible. The authors’ best guess is that these questions were interpreted and mentally rewritten as meaning “is there a *substantial* risk for (harm/cancer/cardiovascular disease/lung disease)” or “is there a risk *large enough to be detected that has been documented*,” rather than “is there *any non-zero* risk, however small.” Accordingly, we interpret agreement with these absolute risk statements as (hesitant or full) endorsement of substantial risk claims, rather than as the rejection of non-zero risk claims. Alternatively, the less emphatic “rather” (dis)agree response alternatives may have been selected to express quantification (e.g., “rather agree with risk x” meaning “yes, some risk, but not a high risk of x”) rather than degrees of certainty (“rather agree with risk x” = “yes, substantial risk of x, but I am not completely confident about this”). Consequently, it is probably safer to interpret these findings cautiously, as reflecting general (negative) attitudes toward the questioned health risks rather than as precise risk estimates.

Almost half of the professionals agreed with the statement that nicotine is a very harmful component of e-cigs. This is a serious misconception in and of itself but probably also represents a major stumbling block for any successful implementation of a THR strategy [26]. Epidemiological data on the long-term use of a related highly-reduced-risk smokeless nicotine product, Swedish snus, reassuringly confirms that any increase in cardiovascular disease or cancer from snus use is speculative, and if it exists is probably about 1 % of that from smoking [27–29]. Likewise, research on classical NRT (patches, inhalers, chewing gum) shows that long-term use of nicotine per se does not reliably increase serious cardiovascular risk [30]. In sum, at least for non-pregnant adults and at the doses typically consumed by vapers, there is little that would justify the professionals’ apparent strong belief in the harmfulness of nicotine as consumed by means of e-cigs [5, 31].

Being contacted in their capacity as health professionals, participants most likely answered the item about the effectiveness of e-cigs as referring to the question whether they believed e-cigs to be effective as smoking cessation aids *when used in the context of a clinical intervention*. More than half of our sample disagreed so, although recent randomized controlled trials, and prospective interventional clinical trials demonstrate that e-cigs can indeed help some people to quit smoking or to substantially decrease their cigarette consumption [17, 18]. In line with this disbelief in the efficacy of e-cigs as quit-smoking aids, in our sample, none (0 %) of the professionals totally agreed recommending e-cigs to their clients/patients, and almost none had information leaflets for potentially interested patients.

Less than 1 out of 10 smokers who want to quit turn for help to a professional; these are probably smokers who have tried many aids and made lots of unsuccessful quit attempts already. If not given the right advice when asking about e-cigs, these “die-hard” smokers, for whom THR might be the best long-term strategy, are denied access to a potentially life-saving alternative. Moreover, health professionals trained as tobacco counselors were even more reluctant about recommending e-cigs than GPs. At the time of the survey, information on the safety or on the effectiveness of e-cigs was completely absent in the training of tobacco counselors. This may, in addition to the almost exclusively negative media communication on e-cigs in Flanders, have contributed to participants’ dismissal of e-cigs as a (clinical) quit-smoking aid.

Even though about two thirds of all participants (correctly, see for example [32, 33]) disagreed that there is a risk of “second-hand exposure” for people in the vicinity of the vaper, results also showed that more than 80 % disagreed with allowing the use of e-cigs in enclosed public places where smoking is prohibited. The majority of our sample also believed that e-cigs will cause renormalization of smoking and that e-cigs will lead to an uptake of conventional smoking, comparable to recent findings from Tan et al. [25] showing that more than half of their nationally representative sample of US adults agreed that e-cigs tempt non-smoking youth to start smoking regular cigarettes and that e-cigs make smoking more acceptable to youth. Available data do not allow to validly support but neither to convincingly exclude the existence of a “gateway-effect” (that is, non-smokers starting to smoke as a consequence of first having started to vape) [11, 34, 35], nor do they suggest an increase in smoking prevalence in populations where vaping prevalence has increased recently, e.g., [36–38] for recent data on the USA and UK smoking and vaping prevalence].

## Conclusions

Our results suggest that few smokers consulting health professionals in Flanders will get medical advice to switch to e-cigs. The virtual absence in the education of GPs as well as in the training of tobacco counselors of up-to-date and balanced summaries of current scientific knowledge on THR in general and on e-cigs in particular, may—at least partially—explain this observation.

## Abbreviations

CASAA, consumer advocates for smoke-free alternatives association; CO, carbon monoxide; GP, general practitioner; e-cigs, e-cigarettes; NRT, nicotine-replacement therapy; RCT, randomized control trial; THR, tobacco harm reduction

## Footnotes

1 In order to put some of these data into perspective, the results obtained from a convenience sample of members of the general public (*n* = 82; male/female ratio = 1:2; *M* age = 26; 23 % smokers; 1 % vapers; 15 % tried e-cigs; recruited via social media (Facebook) on which a call was launched to participate in a web-based survey “to gain more insight into the knowledge and attitudes regarding e-cigs in different populations”) who filled out the same online questionnaire (excluding the items specific to the professional-client interaction) can be summarized as follows. Just like the professionals, the majority (7 in 10) of non-professionals endorsed the statements that e-cigs are harmful to the vaper, whereas 4 out of 10 believed that e-cigs cause harm to people in the vicinity of the vaper; moreover, about 1 in 3 disagreed that e-cigs are healthier than tobacco cigarettes. In the same vein, the majority of the non-professionals also agreed with the statements expressing cancer, cardiovascular, and lung risk of using e-cigs (6 to 7 out of 10), while at the same time, a majority (about 7 out of 10) believed the comparative risks to be lower than those of smoking. If anything, both absolute and relative health risk perceptions were more negative in the non-professional convenience sample than in the professionals. About 3 out of 4 agreed that nicotine is a very harmful component of e-cigs, and similar to the professionals almost 9 out of 10 agreed upon the addictive effect of nicotine-containing e-cigs (1 out of 3 for e-cigs without nicotine). Finally, the majority of the non-professionals also supported a public vaping ban (7 out of 10) and prohibition of sales to minors (8 in 10) and believed that e-cig will lead to renormalization (5 out of 10) and uptake of conventional smoking (7 out of 10) but disagreed that e-cigs are an effective smoking cessation aid (4 in 10). If anything, beliefs in smoking-enhancing consequences (renormalization and uptake smoking) and endorsement of restrictive policies on the use of e-cigs (public vaping ban and prohibition of sales to minors) were somewhat less pronounced in the non-professional sample than in the professionals.
